# Rotavirus vaccination and intussusception: a paradigm shift?

**DOI:** 10.1080/21645515.2020.1770035

**Published:** 2020-06-23

**Authors:** Volker Vetter, Priya Pereira, Bernd Benninghoff

**Affiliations:** GSK Vaccines, Wavre, Belgium

**Keywords:** Rotavirus, gastroenteritis, intussusception, bowel obstruction, vaccination

## Abstract

Rotavirus (RV) is one of the leading causes of severe childhood gastroenteritis in children <5 years of age. Several countries have successfully implemented vaccination against RV disease; however, hesitancy to include RV vaccination in the national immunization program exists and relates, among other reasons, to the results of international post-licensure studies of RV vaccines that established an increased risk of intussusception (IS) in infants following immunization. IS is one of the major causes of bowel obstruction in infants between 4 and 10 months of age. Some studies have investigated the etiology of IS, including the role of natural RV infection and available evidence suggests that RV disease may be an independent risk factor for IS. In this regard, the benefit-risk profile of RV vaccination, which is recognized as positive, could potentially turn out to be even more favorable in preventing IS cases triggered by RV disease. However, further research is prompted to quantify the IS risk attributable to RV disease.

Intussusception (IS) is a common cause of bowel obstruction in children, with the peak age of onset between 4 and 10 months of age.^1^ It occurs when a proximal segment of the gastrointestinal tract telescopes in the immediately adjacent segment.^2^ While this acute, serious medical condition is relatively rare, it can result in blood vessel compression and intestinal ischemia, necrosis, and perforation. If not timely recognized or left untreated, IS can be fatal.^2^

The incidence rates of IS hospital admission vary largely across geographical regions.^3^ Between 2002 and 2018, the median (range) annual incidence of IS hospital admissions per 100 000 ranged from 34 (13–56) in Africa to 90 (9–380) in the Western Pacific region for children <1 year of age.^3^ While pediatric IS is usually idiopathic, predisposing factors, such as infections (including adenoviruses and rotavirus [RV]) and anatomical variations, have been identified.^4^

RV is one of the leading causes of severe childhood gastroenteritis in children <5 years of age, accounting for substantial morbidity globally and high mortality in resource-limited countries.^5^ Several countries have successfully implemented vaccination against RV disease, demonstrating reduced cases of severe diarrhea and RV disease requiring hospitalization^6,7^ but also reduced mortality rates.^6^ However, vaccine uptake remains low in many regions, partially due to low priority ascribed to RV gastroenteritis prevention and cost constraints.^8^ Furthermore, hesitancy to implement RV vaccination in the national immunization program relates to the evidence from post-licensure studies that established an increased risk of IS in infants shortly (during the 7-day period) following RV vaccine administration.^9–11^

The first developed RV vaccine, tetravalent rhesus-human reassortant (*Rotashield*; Wyeth Laboratories) was withdrawn post-licensure in 1999, within a year following introduction, due to its association with IS (relative risk of IS was 58.9 [95% confidence interval: 31.7–109.6] post-dose 1 and 11.0 [4.1–29.5] post-dose 2).^10,12^ Following thorough pre-licensure safety evaluation with regard to IS,^13,14^ two second-generation RV vaccines (HRV; *Rotarix*, GSK, and HBRV; *RotaTeq*, Merck & Co., Inc.), have been recommended since 2009 by the World Health Organization (WHO) for worldwide use in routine RV vaccination programs in infants. Albeit lower than for *Rotashield*, accumulated evidence suggests an increased risk of IS during the 7-day period post-dose 1 (5.4 [3.9–7.4, 3 studies] for HRV and 5.5 [3.3–9.3, 3 studies] for HBRV) and to a lesser extent during the 7-day period post-dose 2 (1.8 [1.3–2.5, 4 studies] for HRV and 1.7 [1.1–2.6, 3 studies] for HBRV), in a meta-analysis of 5 post-licensure studies.^10^ These data suggest a class effect for both RV vaccines in terms of IS risk after immunization. Nevertheless, the WHO Global Advisory Committee on Vaccine Safety emphasized that the benefit-risk profile of both licensed RV vaccines remains favorable, with the benefits outweighing the risk of IS.^15^

Of note, the risk of IS attributable to RV vaccination depends on age, and recent evidence suggests that the absolute risk of IS is only slightly increased by vaccination (increased risk attributable to vaccination was 1.7 for HRV and HBRV) when the vaccines are administered within the recommended time window of <3 months.^16–18^ An active surveillance study conducted in seven African countries reported a relative risk of 0.25 (<0.001–1.16) and 0.76 (0.16–1.87) during 1 week post-dose 1 and post-dose 2 of HRV, respectively, when administered in children <3 months of age.^17^ More recently, WHO Global Advisory Committee on Vaccine Safety (GACVS) reviewed data on the safety of HBRV in five sub-Saharan African countries and of neonatal human rotavirus vaccine (nHRV; *Rotavac*) in parts of India, reporting a non-significant increase in IS risk post-dose 1 of HBRV in sub-Saharan Africa and of nHRV in India.^19^ The risk of IS within 1 week after vaccination was 4.11 (0.79–11.52) post-dose 1, 0 post-dose 2, and 0.86 (0.28–1.92) post-dose 3 of HBRV.^19^ For nHRV using self-controlled case-series (SCCS) method on smaller sample size, IRR for the first 7 days post-dose 1, 2 and 3 compared with the period from day 28 to 1 year of age was 0.83, 0.86, and 1.65, respectively. These data were not statistically significant, suggesting no association between nHRV vaccination and IS. Similarly, no significant difference was seen for the 8–21-day risk window after vaccination. Since the limited sample size comparison of products in the same risk window is not accurate, more data are needed to guide safety evaluation of new rotavirus vaccines and across their use in national immunization programs.^19^ These data prompt pediatricians to counsel parents to monitor vaccinated children and to seek medical advice in case signs or symptoms suggestive of IS emerge.

Considering the real-world evidence accumulated since the launch of the two globally available RV vaccines (HRV and HBRV) and the high vaccine coverage in some regions, the increased risk of IS following RV vaccination does not seem to translate into an overall long-term increase of IS in countries with RV vaccination included in their national immunization programs. In Australia, where both *Rotarix* and *RotaTeq* are available, one of the first post-marketing surveillance studies for IS following vaccination revealed no overall increase in IS following receipt of RV vaccine although some evidence of an elevated risk following the first dose was observed. The authors suggest that any increased risk after the first dose is compensated by a reduced risk after later doses.^20^ To unravel whether the observed association between RV vaccination and IS translates into a higher rate of IS-related hospitalizations, Tate et al.^21^ examined retrospectively pre-(2000‒2005) and post-RV vaccination (2007‒2013) IS rates in the United States. No change in IS hospitalization rates was observed among children <12 months of age, but the rate in children 8 to 11 weeks was significantly elevated in all post-vaccination years, except in 2011 and 2013 (Table 1).

**Table 1.** Overview of calculated IS hospitalization rates recorded before and after the introduction of annotated rotavirus vaccinations into different immunization programs. All data are derived from Retrospective Analysis Studies

			IS incidence		IS hospitalization			
			{Pre-vaccination era}	{Post-vaccination era}	{Pre-vaccination era}	{Post-vaccination era}		
Country	Vaccine	Age	Cases per 100,000	IRR (95% CI)	Cases per 100,000	Cases per 100,000	RR (95% CI)	Reference
US	*Rotashield*	<12 m	NR	NR	45.0–31*	31*	NR	Simonsen et al., 2001^22^
US	HBRV	6–14 w	NR	NR	10.2	12.0–15.8	1.18 (0.92–1.54) – 1.55 (1.22–1.57)	Yen et al., 2012^23^
		15–24 w	NR	NR	39.9	34.7–42.1	0.87 (0.75–1.00) – 1.05 (0.92–1.20)	
US	HRV/HBRV	6–14 w	NR	NR	15.0 (12.6–17.8)	17.0–22.5	1.13 (0.90–1.43) – 1.50 (1.22–1.83)	Tate et al., 2016^5^
		15–24 w	NR	NR	46.4 (38.2–50.8)	41.8–55	0.90 (0.79–1.03) – 1.18 (1.05–1.34)	
Canada	HRV/HBRV	<12 m	23.4 (21.5–25.4)	0.96 (0.78–1.18)	~20.0–27.5	~22.5–30	NR	Hawken et al., 2017^24^
Taiwan	HRV/HBRV	6–14 w	77.0 (for <12 m)	NR	26.2 (20.0–34.4)	22.5 (17.1–29.6)	0.86 (0.58–1.26)	Yen et al., 2017^25^
		15–24 w	NR	NR	79.8 (68.8–92.5)	66.3 (57.0–77.2)	0.83 (0.67–1.03)	
Korea	HRV/HBRV	<12 m	234.1	0.66–0.81^b^	NR	NR	NR	Cho et al., 2018^26^
		8–11w^a^	66.6	0.29–0.76	NR	NR	NR	
		6–14w^a^	NR	0.44 (0.35–0.55)	NR	NR	NR	
		15–24w^a^	NR	0.60 (0.53–0.67)	NR	NR	NR	

IS, intussusception; IRR, incidence rate ratio; RR, rate ratio; 95% CI, 95% confidence interval; HRV, monovalent human rotavirus vaccine (*Rotarix*); HBRV, pentavalent bovine rotavirus vaccine (*Rotateq*); Age, age at which children were vaccinated; m, months; w, weeks; NR, not reported; ^a^, calculated annual incidence rate; ^b^, data for the entire post-vaccination era; * Rate calculated per 100,000 infant years. For the study citing *Rotashield* data, post-vaccination era data are reported only for the year 1998–1999, when *Rotashield* was available in the United States.

The observation of no sustained population-level change in overall IS hospitalization rates is in line with previous studies conducted in the US.^23,27,28^ These studies reported no consistent change in IS hospitalization rates after vaccination when compared to pre-vaccination era levels despite increasing RV vaccine coverage in US infants over the period under evaluation (Table 1).^23,27,28^ More specifically, Yen et al.^23^ noted an increase in IS hospitalization rates among infants aged 8–11 weeks (to whom most first doses of RV are given) but also observed that IS hospitalization rates among older infants tended to be lower in post-vaccination years compared with pre-vaccination years, although these differences were not statistically significant (Table 1). As for the study in Australia, the authors expressed the need to confirm whether later in infancy IS hospitalization rates are decreased in vaccinated infants and whether this decline could offset, at population level, any short-term increase in IS after vaccination.

A retrospective study conducted in Canada showed no increase in the incidence of IS-related hospital admissions after the introduction of routine RV immunization programs.^24^ Besides, despite a much higher risk of IS with *Rotashield* when compared to the currently available RV vaccines, ecological assessments of *Rotashield* did not reveal enhanced infant IS admissions during the *Rotashield* era.^22^ The authors reported that the overall risk of *Rotashield*-related IS hospital admissions was considerably lower than previous estimates based on the immediate post-vaccine period.^22^ Moreover, a recent longitudinal cohort study of commercially insured US children provides supporting evidence for an overall reduced risk of IS in RV-vaccinated children. In this study, a non-significant decrease in IS was found in fully RV-vaccinated children followed up to the age of 2 years.^29^ Similar results were obtained in a study conducted in Taiwan, where mean IS hospitalization rates were lower during the post-vaccination period for children aged <12 months with the greatest decline among children aged 25–34 weeks, although the difference with pre-vaccination period did not reach statistical significance (Table 1).^25^ This observation is in agreement with a recent report from South Korea documenting a decline in the incidence of IS in infants after the introduction of the RV vaccine (Table 1).^26^

Considering the increased risk of IS following RV vaccination in birth cohorts, an impact on long-term IS incidence rates in vaccinated children would be expected over successive years. The reasons for the absence of such observations are likely to be multifactorial; however, the findings by Willame et al.^30^ in the current edition of this journal may offer a partial explanation. Given the observed association between RV vaccination and IS, the authors explored the role of natural RV infection in the development of IS in infants below 1 year of age through a retrospective, self-controlled case-series analysis. They observed a positive association between RV gastroenteritis and IS using US insurance claims data. An increased risk of IS after RV gastroenteritis was observed in the main analysis, where the calculated risk factor was 79.6 (95% CI: 38.6–164.4) in the 7-day period and 25.5 (13.2–49.2) in the 21-day period. Notably, also the sensitivity analysis showed an increased risk of IS after fracture during the same periods (6.1 [3.0–12.7] and 2.8 [1.5–5.4]), which was an unrelated event used to evaluate the quality of the claims data to address this research question. To refine the data and to adjust for potential confounding detected in the main analysis, a post hoc analysis was performed, which still suggested an association between RV gastroenteritis and IS, but not between fracture and IS (see ref. Willame et al.^30^). Due to limitations inherent to the study (e.g. diagnosis uncertainty, true disease onset possibly misclassified, potential unknown bias, and confounding), the risk could not be robustly quantified but is consistent with previous reports suggesting that RV gastroenteritis could constitute a risk factor for IS.^31–34^

The question remains how to reconcile results from post-marketing studies (showing an increased risk of IS following RV vaccination, as reflected in the labels of the two RV vaccines) with long-term data on IS incidence rates in countries with implemented RV vaccination. It can be argued that while RV vaccination has been associated with an increased risk of IS, disease prevention through vaccination may have averted a substantial number of IS cases caused by natural RV infection that would have occurred later in childhood.

The study by Willame et al.^30^ sheds new light on the risk of IS due to naturally occurring RV and on the implication of these findings for RV vaccination programs. Indeed, the pivotal question is centered around the quantification of the IS risk attributed to RV disease, and how to weigh it against the increase of IS following RV vaccination, in a situation of optimal vaccine coverage and early completion of the vaccination schedule. In this regard, the benefit-risk profile of RV vaccination, which is recognized as positive, could potentially turn out to be even more favorable in preventing IS cases triggered by RV disease. Area-specific factors, such as varying IS background incidence rates, could critically impact the benefit-risk assessment, with most likely tangible effects in areas experiencing the highest background rates of IS (Figure 1). In this context, one may anticipate that this effect of RV vaccination would add to the multiple indirect effects attributed to RV vaccination, beyond reducing RV gastroenteritis-related mortality and morbidity (e.g. community protection through herd effect, reduction of RV nosocomial infections, reduced incidence of childhood seizures).^37^

**Figure 1.**
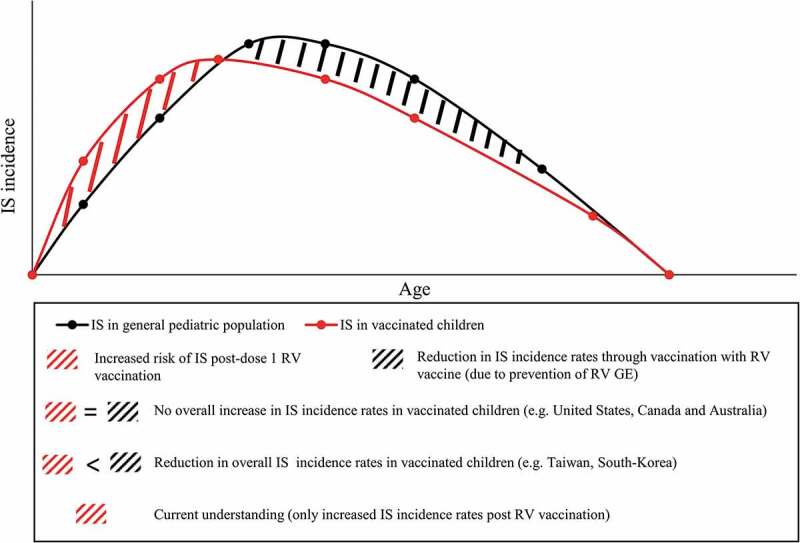
Schematic presentation of IS incidence rates occurring in the general pediatric population (in concordance with studies such as Tate et al.^35^ and Tai et al.^36^) and hypothetical IS incidence rates in children vaccinated against RV disease. IS, intussusception; RV, rotavirus; RV GE, rotavirus gastroenteritis

This recent new perspective on RV vaccination, which also includes its public health impact, might be critical in motivating health-care professionals and caregivers to encourage vaccination in general and in convincing policy-makers to further implement early RV vaccination into national immunization program. To support those assumptions, which should still be taken with caution, future research to quantitatively assess the IS risk attributed to RV disease is prompted, as well as generation of disease surveillance data, a fundamental aspect of the continuous evaluation of IS risk in vaccinated pediatric populations.

## Trademark statements

*Rotarix* is a trademark owned by the GSK group of companies. *RotaTeq* is a registered trademark of Merck&Co., Inc. *Rotavac* is a trademark of Bharat Biotech.
